# Penile Varicose Vein in *Akita inu*, 7-Year-Old Dog: A Clinico-Pathological Study

**DOI:** 10.3390/vetsci9020047

**Published:** 2022-01-27

**Authors:** Simona Attard, Luisa Vera Muscatello, Giuseppe Mazzullo, Maria Carmela Pisu

**Affiliations:** 1 VRC—Centro di Referenza Veterinario, 10138 Torino, Italy; simonattard@libero.it; 2 Department of Veterinary Medical Sciences, University of Bologna, 40064 Ozzano dell’Emilia, Italy; luisaver.muscatello2@unibo.it; 3 MyLav La Vallonea, Laboratorio di Analisi Veterinarie, 20017 Milan, Italy; 4 Department of Veterinary Sciences, University of Messina, 98168 Messina, Italy; giuseppe.mazzullo@unime.it

**Keywords:** genital system, vascular disease, estrogen receptors, surgery

## Abstract

Penile varicose veins are a rare lesion described in human medicine. A seven-year-old intact *Akita inu* male dog presented with a red penile neoformation. It was further referred for a specialist andrological examination. During the visit, ultrasonography of genital apparatus and cytology of the penile lesion were performed. A suspected neoformation of vascular origin was observed on ultrasonography. Cytology was inconclusive as composition of the sample revealed blood elements only. The neoformation was surgically removed and the excised sample was submitted for the histological examination, which revealed an anomalous varicose dilatation of the venous vascular structures. In human medicine, varicose veins are part of the spectrum of chronic venous disease and include spider telangiectasia, reticular veins, and true varicosities. Reports of penile localization of varicose veins is rare in human medicine and, to the best of our knowledge, it has never been reported in veterinary medicine.

## 1. Introduction

Varicose veins represent a chronic pathology of the venous vessels [1] due to reduced smooth muscle cell contractability of the walls of the vessels [2].

In human medicine, the pathology is well-known and well-studied: varicose veins belong to the group of the chronic venous diseases. Generally, it is more common in older women with venous insufficiency and high concentrations of estrogen in the blood. 

Varicose vein transformation is a minor phenomenon of undifferentiated connective tissue dysplasia (UCTD) leading to failure of contractability of their walls due to abnormalities in the fibrous structures and extracellular matrix [2]. 

In animals, varicose veins are rare, with few case reports that described an ocular localization in dog [3,4].

In the genital organs, varicose veins have been described in some cases of vaginal changes in mares, and never in males [5].

The aim of this study was to report the clinical and pathological findings of a penile varicose vein in a dog.

## 2. Case Description

### 2.1. Clinical Findings

**Figure 1:**
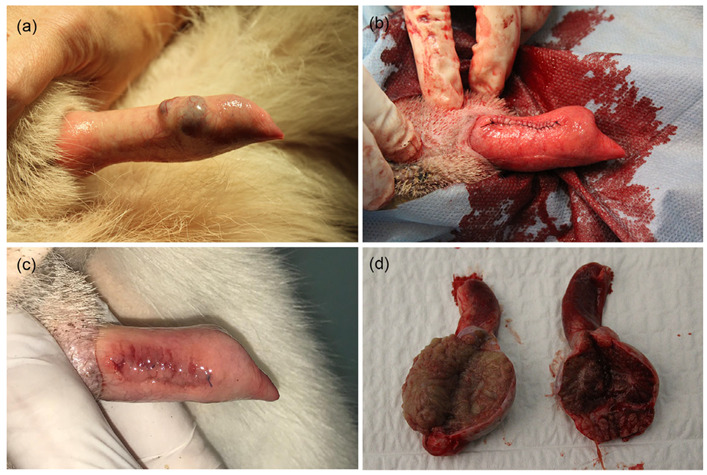
Clinical evaluation of penile lesion. (**a**) Appearance of the injury on the unsheathed penis. (**b**) Appearance of the lesion during blood engorgement. (**c**) Appearance of the surgical wound on the glans penis at follow up. (**d**) Appearance of the testicles cut longitudinally.

A seven-year-old *Akita inu* male dog was presented because of an unusual swelling which appeared on the glans penis, particularly evident during moments of excitement. The pet was not used for breeding purposes. The swelling, slightly detected on the surface of the organ on the ventrolateral left side, appeared ovoidal in shape, 0.9 × 0.6 cm, with irregular margins blurring into the surrounding tissue, and with a hollow center. The swelling was not painful and showed the tendency to engorge with blood when a slight pressure was applied on the organ (Figure 1a). The owner had noticed the lesion almost two months prior, and the routine tests conducted were within the normal range. The lesion did not undergo any remarkable change since the first evidence.

The scrotum was normal in conformation and appearance. Testicles were within the scrotal sack. Symmetry, size, and mobility were maintained. On palpation, the right testicle was soft in consistency, while the left one was firm. The foreskin was normal. Ultrasound examination of the penile lesion showed an oval outline and a heterogeneous eco-structure. There were vascular gaps with low resistor type limited peripheral afferent vascularity. Cavernous arteries had a rate of systolic pressure of 45 cm/s. The urethra was patent, without lesions. 

The left testicle was in place, with normal eco-structure. The right testicle was in situ, with a decreased size compared to the contralateral with altered eco-structure showing hypoechoic areas with faded edges. 

The prostate was evaluated by ultrasound and was 4 × 3 cm in size, with a normal eco-structure and without focal lesion.

Fine needle aspiration cytology (FNAC) of the lesion was performed and the smears were stained with May–Grunwald–Giemsa Quick, revealing only a blood-filled background without any cellular details; hence, it was inconclusive. Ultrasonography of the genital apparatus was performed using a linear 12 MHz probe (Mylab 50, Esaote, Genoa, Italy).

Based on the obtained results, anesthesia for excisional biopsy and orchiectomy were performed. The anesthetic protocol provided intramuscular premedication with dexmetedomidine (2 μg/kg, Detroquillan Esteve, Barcelona, Spain) and methadone (0.2 mg/kg, Semfortan, Dechra, Northwich, UK). Induction was performed with propofol (4 mg/kg intravenous, Proposure, Merial Italia, Milano, Italy) and maintained with 2% isoflurane inhalation anaesthesia (Isoflo, Zoetis, Parsippany, NJ, USA). 

Orchiectomy was performed by prescrotal approach, and the penile lesion was excised for excisional biopsy (Figure 1b); careful hemostasis was carried out and the surgical breach was sutured with a polydioxanone 4-0 suture. 

Post-operative follow-up did not reveal any complications. The surgical wound healed uneventfully (Figure 1c). The testicles were grossly normal to slightly atrophic (Figure 1d). 

The excised penile tissue and both testes were fixed in 10% buffered formalin and sent to the laboratory for histological examination. 

### 2.2. Histopathological and Immunohistochemical Studies 

**Figure 2:**
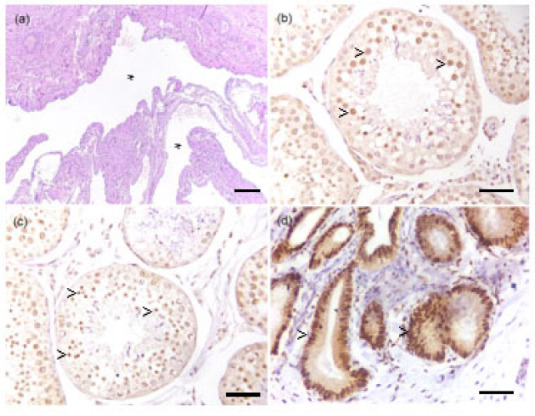
Histological evaluation of penile lesion and immunohistochemistry for the estrogen receptor (ER) on testicles. (**a**) Ectatic vessels (asterisks), covered by flattened monolayered endothelium without atypical features, hematoxylin and eosin, 40×, bar 200 micron. (**b**) Testicle of the case, ER immunohistochemical nuclear expression (arrowheads) in both Sertoli cells and Leydig cells, but not in the germ cells, 400×, bar 100 micron. (**c**) Control testicle, ER immunohistochemical nuclear expression (arrowheads) in both Sertoli cells and Leydig cells, but not in the germ cells, 400×, bar 100 micron. (**d**) Control uterus, ER immunohistochemical nuclear expression (arrowheads) in the uterine glands and myometrium, 400×, bar 100 micron.

In the left testicle there were no pathological findings. In the right testicle, the parenchyma was entirely composed of seminiferous tubules in which the absence of mature germ cells was observed. Seminiferous tubules were covered by Sertoli cells, often vacuolized (degeneration), mixed with occasional germ cells, such as spermatogonia and spermatocytes, in the early stages of maturation (compatible with spermatogenic arrest). The histopathological picture was suggestive of diffuse testicular tubular atrophy. In the tissue sample from the glans penis (size of 2 × 1 × 0.5 cm), multifocal vascular structures were observed, coalescing, some of which contain red blood cells, markedly dilated, covered by flattened well-differentiated endothelial cells without any pathological alteration (Figure 2a). In the adjacent stroma there were multifocal perivascular aggregates of rare lymphocytes and plasma cells. The mucosal epithelium of the glans revealed multifocal hyperplasia. The lesion was excised with a close but clean deep margin (<1 mm). The histopathological picture was compatible with marked, multifocal vascular dilatation. In the analyzed sample no elements indicating neoplasia were observed. The histological features were overall interpreted as an anomalous varicose dilation of venous vascular structures.

Immunohistochemistry for ER (estrogen receptor) was performed on formalin-fixed paraffine-embedded tissue sections of the testicles. Antigen retrieval was obtained with citrate buffer pH 6.0 for 10 min at 750 W in microwave. Primary antibody (ER, polyclonal, ThermoFisher Scientific, Waltham, MA, USA, dilution 1:100) was incubated overnight at 4 °C. ABC kit (Vector) was used to amplify the reaction. Two normal testicles and a normal canine uterus were used as positive control.

A moderate intense diffuse nuclear positive staining was observed in both Sertoli cells and Leydig cells, but not in the germ cells of all the examined testicles (Figure 2b). No differences were detected across the case and controls (Figure 2c). A diffuse intense endometrial and myometrial nuclear staining was detected in the positive control (Figure 2d).

## 3. Discussion

This case report is the first case of a penile varicose vein in dog, which has not been described previously in veterinary medicine literature. Varicose veins are most frequently described in human medicine, where they are part of the spectrum of chronic venous disease. Generally, it is more common in women and in the older age group with venous insufficiency, including venous ulceration [1]. The risk factors are family history of varicose veins, smoking, pregnancy, use of the oral contraceptive pill, deep venous thrombosis, lower limb plaster cast, long period of standing, or previous venous surgery [6]. The higher prevalence in females has been linked to the high concentration of blood estrogen levels [7,8]. Varicose vein transformation is considered to be a minor phenomenon of undifferentiated connective tissue dysplasia (UCTD) leading to failure of contractability of their walls due to abnormalities in the fibrous structures and extracellular matrix [2]. In animal models, the concentrations of neutrophils, monocytes, macrophages, and lymphocytes and levels of matrix metalloproteinase increase in venous valves exposed to high pressures for prolonged periods of time. Over time, the venous valves, exposed to high pressures, demonstrate adverse remodeling with decreases in leaflet length and thickness. Turbulent flows may promote inflammatory and prothrombotic changes, possibly causing the loss of structural integrity of the vein wall [1]. Furthermore, in recent studies it has been observed that, around varicose valves, there are infiltrations of monocyte/macrophages, alterations in the ratios of collagen subtypes, matrix metalloproteinase variations, and differences in rates of apoptosis. However, it is difficult to demonstrate if those conditions are the cause or the effect of the lesions [6]. 

Glans localization, to the best our knowledge, has never been described before in veterinary medicine. In men, this is also a rare event, frequently correlated with other pathologies of hormonal dysfunction. A case report of syndromes of functional hypogonadotropic hypogonadism and sterility in males with elevated serum estradiol (56 pg/mL—normal range 8–38 pg/mL) has been described. A 36-year-old male was a genotypic male, but phenotypically, he exhibited signs of long-standing estrogen excess, such as a feminine body build, gynecomastia, and varicose veins. In that case, a significantly elevated serum estradiol level was reported and considered the most likely cause of a concurrent functional suppression of gonadotropin secretion [9]. The clinical signs were comparable with those of the dog in the present case report. The patient was a pet and his owner refused to allow any supplementary examinations.

In humans, a strong relationship has been assessed between hyperestrogenism and varicose veins [9]. The effect of estrogen on the risk of varicose veins may explain, in part, the increased prevalence among women [1]. Unfortunately, serum estrogen concentration of the patient could not be estimated in the present case, but immunohistochemistry on tissue sections of the testicles was performed to investigate the estrogen level, which was in accordance to the findings of other research [9]. Estrogen receptors (ERs) have been reported in the male reproductive system of several species, including humans, goats, mice, monkeys, rabbits, rats, and roosters [10]. However, the distribution of ERs is not uniform across species, and studies in some species were performed before the discovery of the second ER isoform, the estrogen receptor β. Although estrogen receptor α (ER α) and estrogen receptor β (Erβ) are similar in structure, they differ in C-terminal ligand binding and in N-terminal transactivation domains. [10]. Estrogen is mainly produced in the adipose tissue, but its receptors (ERα and ERβ) are localized in most cell types of the body including the testes, which suggests an important role for estrogen in regulating testicular cell function and reproductive events. Conflicting data is reported about the expression of ERα in the testes, and few studies have been conducted examining the localization of ERα-immunoreactive structures in the testes. It has been reported that ERα is present in the Sertoli cells of multiple species including boars, cats, humans, pigs, and rodents. ERα immune reactivity was observed in the interstitial space of seminiferous tubules. Based on their location, ERα immunoreactive structures were thought to be within the Leydig cells of testis [11]. In the cryptorchid testis, ERα immunoreactivity was detected in the basal part of seminiferous tubules, as well as in the interstitial space of tubules [11]. These cells are judged to be Sertoli cells and Leydig cells, respectively, based on their morphology.

ERα immunoreactivity in the cryptorchid testis was significantly increased compared to the control testis [11]. Multiple cell types of dog and cat testes stained positive for ERβ. In the rete testis and efferent ductules, epithelial cells were weakly positive for ERβ. Most epithelial cells of the epididymis and the vas deferens exhibited strong positive staining in both species. In addition, double staining was used to demonstrate colocalization of both ERα and ERβ in efferent ductules of both species [10]. 

In our case, ER immunohistochemical expression was comparable between the testicle of the affected subject and the control testicles. This does not allow definitive correlation of hyperestrogenism to the varicose veins in this case. 

## 4. Conclusions

The case described represents, to our knowledge, the first case described in Veterinary Medicine to date, and specifically for the canine species. The present case report offers information regarding the incidence of varicose veins in dogs, their location, evolution, and related etiopathogenetic aspects. The varicose vein is a pathological process that does not predispose neoplastic evolution, therefore the possibility of monitoring the lesion without surgery may be a therapeutic option. 

## Data Availability

The data presented in this study are available on request from the corresponding author.
